# Accelerated Growth Plate Mineralization and Foreshortened Proximal Limb Bones in Fetuin-A Knockout Mice

**DOI:** 10.1371/journal.pone.0047338

**Published:** 2012-10-16

**Authors:** Jong Seto, Björn Busse, Himadri S. Gupta, Cora Schäfer, Stefanie Krauss, John W. C. Dunlop, Admir Masic, Michael Kerschnitzki, Paul Zaslansky, Peter Boesecke, Philip Catalá-Lehnen, Thorsten Schinke, Peter Fratzl, Willi Jahnen-Dechent

**Affiliations:** 1 Department of Biomaterials, Max Planck Institute of Colloids and Interfaces, Potsdam, Germany; 2 Department of Osteology and Biomechanics, University Medical Center Hamburg-Eppendorf, Hamburg, Germany; 3 Helmholtz Institute of Biomedical Engineering, Biointerface Laboratory, Rheinisch-Westfälische Technische Hochschule (RWTH) Aachen University, Aachen, Germany; 4 Beamline ID2, European Synchrotron Radiation Facility, Grenoble, France; Georgia Health Sciences University, United States of America

## Abstract

The plasma protein fetuin-A/alpha2-HS-glycoprotein (genetic symbol *Ahsg*) is a systemic inhibitor of extraskeletal mineralization, which is best underscored by the excessive mineral deposition found in various tissues of fetuin-A deficient mice on the calcification-prone genetic background DBA/2. Fetuin-A is known to accumulate in the bone matrix thus an effect of fetuin-A on skeletal mineralization is expected. We examined the bones of fetuin-A deficient mice maintained on a C57BL/6 genetic background to avoid bone disease secondary to renal calcification. Here, we show that fetuin-A deficient mice display normal trabecular bone mass in the spine, but increased cortical thickness in the femur. Bone material properties, as well as mineral and collagen characteristics of cortical bone were unaffected by the absence of fetuin-A. In contrast, the long bones especially proximal limb bones were severely stunted in fetuin-A deficient mice compared to wildtype littermates, resulting in increased biomechanical stability of fetuin-A deficient femora in three-point-bending tests. Elevated backscattered electron signal intensities reflected an increased mineral content in the growth plates of fetuin-A deficient long bones, corroborating its physiological role as an inhibitor of excessive mineralization in the growth plate cartilage matrix - a site of vigorous physiological mineralization. We show that in the case of fetuin-A deficiency, active mineralization inhibition is a necessity for proper long bone growth.

## Introduction

Fetuin-A/alpha2-HS-glycoprotein (genetic symbol *Ahsg*) is a 52 kDa glycoprotein member of the type 3 cystatin-family proteins composed of two N-terminal cystatin protein domains, and a third C-terminal domain rich in proline [1]. Fetuin-A has originally been described as the most abundant globulin in fetal calf serum [2], and was long known as one of the most abundant non-collagenous proteins [3] highly enriched in the mineralized bone matrix [4]–[7]. Fetuin-A has a high affinity mineral binding site located in the amino-terminal domain D1 enabling it to inhibit ectopic calcification [8], [9] by forming soluble colloidal nanospheres [8], [10]. These colloids are variously termed calciprotein particles CPPs in analogy to lipoprotein particles [9], [11], or fetuin-mineral complex FMC [10] or nanons [12]. Soluble protein-mineral complexes containing fetuin-A are regarded as physiological byproducts of mineral metabolism preventing pathological calcification at sites of high mineral supersaturation [13], [14]. Complexes of fetuin-A, albumin and calcium phosphate have been detected serum [15] including human serum of chronic kidney disease patients [16], [17] and in ascites of patients with peritoneal calcifying sclerosis [18]. These complexes have been previously implicated as the source of an entity dubbed “nanobacteria” that have vexed the microbiology and biomineralization community for almost a decade [12], [14], [15], [19]. Thus the role of fetuin-A as an important regulator of mineralization or a “mineral chaperone” [13], [20] has gained abundant support, but its role in mineralization control of the tissue which is most abundant of it – bone – is understudied. When fetuin-A deficient mice (*Ahsg*−/−) became available [21] bone growth and remodeling phenotypes were examined in these mice. The skeletal structure of *Ahsg*−/− mice appeared normal at birth, but abnormalities were observed in adult *Ahsg*−/− mice. Maturation of growth plate chondrocytes was impaired, and femurs lengthened more slowly between 3 and 18 months of age in *Ahsg*−/− mice. Previously it had been found that fetuin-A is a soluble transforming growth factor-beta (TGF-beta)/bone morphogenetic protein (BMP)-binding protein controlling cytokine access to membrane signaling receptors [22], [23]. Hence the altered bone phenotype was explained in terms of failure to block TGF-beta-dependent signaling in osteoblastic cells. Mice lacking fetuin-A displayed growth plate defects, increased bone formation with age, and enhanced cytokine-dependent osteogenesis [24]. Tumorigenesis experiments employing *Ahsg*−/− mice further supported the hypothesis that fetuin-A is an antagonist of transforming growth factor beta in vivo, in that it inhibited intestinal tumor progression. All these experiments had been performed using the first available fetuin-A deficient mouse strain 129,B6*Ahsg* ^tm1mbl^, which had a mixed 129Sv×C57BL/6 genetic background [21]. We created two more strains of fetuin-A deficient mice with defined genetic backgrounds (B6-*Ahsg^t^* ^m1wja^, D2-Ahsg^tm1wja^) by backcrossing to C57BL/6 and DBA/2 mice. Surprisingly all D2-*Ahsg* ^tm1wja^ mice showed massive soft tissue calcification throughout their body, while B6-*Ahsg* ^tm1wja^ mice did not [25]. The latter mice calcified when additionally challenged with heminephrectomy and high mineral diet [26], [27]. These results showed that fetuin-A is a systemic, soluble inhibitor of pathological mineralization that is backed up by other genetic factors rendering mouse strains prone to or resistant to dystrophic calcification. Integrative genomics of the so-called *Dyscalc* locus led to the identification of *Abcc6* as the major causal gene for dystrophic cardiac calcification [28]. Abcc6 deficient mice develop soft tissue calcifications that seem to be less extensive than in D2-*Ahsg* ^tm1wja^ mice both in localization and extent. Interestingly, Abcc6 deficiency is associated with reduced plasma fetuin-A levels and the calcifications can be partially corrected by over-expressing fetuin-A [29] suggesting that fetuin-A acts downstream or in concert with Abcc6. Despite heavy early onset and life-long progressing dystrophic calcification in Abcc6 and especially in DBA/2 fetuin-A deficient mice, true osteogenesis has never been reported in these mice. This contrasts the popular view that pathologic calcification is necessarily a form of ectopic osteogenesis. More likely both genes seem to be involved in systemic mineral homeostasis and transport. Therefore we have termed fetuin-A a mineral chaperone mediating the solubilization, transport and elimination from circulation of otherwise insoluble mineral much like apolipoproteins help in lipid transport and metabolism [13], [20].

*In vitro* experiments involving re-mineralization of decalcified bone tissue showed that in the absence of fetuin-A, extra-fibrillar mineralization of the collagen fibrils can readily occur, but proper collagen fibril mineralization cannot [30], [31]. Recent potentiometric titration investigations of fetuin-A mediated mineralization in solution found resulting mineral in the form of HAP mineral platelets. We asked if the lack of fetuin-A in bones of *Ahsg*−/− mice would indeed result in altered bone quality. Here, we report a detailed multiscale analysis of bones from fetuin-A deficient and fetuin-A producing mice. Surprisingly, contrary to expected differences at the materials level, all micro-mechanical and micro-structural parameters were found to be similar in *Ahsg−/−* and *Ahsg+/+* bone. In sharp contrast, macro-mechanical strength and bone length were both altered in *Ahsg−/−* mice. This phenotype was associated with enhanced growth plate cartilage mineralization corroborating the role of fetuin-A as a blood-borne systemic inhibitor of mineralization and thus, of pathological calcification in the extracellular and vascular compartments.

## Materials and Methods

### Animals and Diets

The animal welfare committee of the Landesamt für Natur-, Umwelt- und Verbraucherschutz LANUV of the state of North Rhine Westfalia approved our animal study protocol. Animal maintenance and handling was according to the Federation for Laboratory Animal Science Associations FELASA recommendations. Animals were sacrificed by isoflurane overdosing. We studied fetuin-A deficient mice (B6-*Ahsg* ^tm1wja^, N11 backcross generation) and wildtype littermates. Mice were maintained in a temperature-controlled room on a 12-hour day/night cycle. Food and water were given ad libitum. Fetuin-A deficient *Ahsg*−/− mice on a pure C57BL/6 genetic background were analyzed using adult mice with ages between 4 and 12 months for histological and microstructural analyses.

*Histomorphometry and mechanical testing-* After the initial analysis by contact X-ray (Faxitron Xray Corp., Lincolnshire, USA) the vertebral bodies L2 to L5 were dehydrated and embedded non-decalcified into polymethylmetacrylate (PMMA) for sectioning. Sections were either stained with toluidine blue or by the von Kossa/van Giesson procedure as described [32]. Static and cellular histomorphometry was carried out using the OsteoMeasure system (Osteometrics, Decatur, USA) following the guidelines of the American Society of Bone and Mineral Research [33]. The cortical thickness of femora was quantified by µCT scanning using a µCT40 (Scanco Medical, Switzerland). Femora were then tested to failure by three-point bending on a servohydraulic device (Z.2.5/TN1S, Zwick/Roell, Ulm, Germany) as described [34].

### Sample Preparation for High-resolution Microstructure Analysis

Bone samples were sectioned from the mid-diaphysis of femora from 12-month-old mice. The femora were sectioned along the tangential-longitudinal plane (Figure S1). Each half of the femoral cortex was polished mechanically from both sides in an automatic polisher (Logitech PM5, Logitech Ltd., Glasgow, UK) until the sample was planar. Care was taken to make sure the samples’ longitudinal axes were parallel to the main longitudinal bone axis. Subsequent polishing was performed using 5, 3, 1 µm grit-sized diamond particles (DP-Spray P, Struers A/S, Ballerup, Denmark), in that order, until the final sample thickness was 50 µm. Gross sectioning of cortical bones was performed with a slow speed saw (Isomet Low Speed Saw, Buehler Ltd., Lake Bluff, IL, USA) and further processed by UV laser micro-dissection prior to spectroscopic and micro-mechanical probing of the tissues. The samples were further sectioned in an UV laser micro-dissection system (PALM MicroBeam C, P.A.L.M. Microlaser Technologies GmbH, Bernried, Germany) [35] to isolate sections that were 150 µm (width) ×50 µm (thickness) ×2.5 mm (length) in dimension. All sectioning and micro-dissection were performed on hydrated samples. Further detail of high-resolution microstructural analysis is given in supporting methods appendix.

### Optical Microscopy of Microstructural Features in the Bulk Material

Macroscopic structural features in both *Ahsg*+/+ *and Ahsg*−/− were observed with a polarized microscope (Leica DM RXA2, Leica Microscopy and Systems GmbH, Wetzlar, Germany) equipped with a Leica DFC digital camera. Specifically, in reflective mode the surface of the samples were examined to compare texture as well as osteocyte lacunae morphology and orientation.

### Scanning Electron Microscopy of Microstructural Features in the Bulk Material

The bulk microstructure of *Ahsg*+/+ and *Ahsg*−/− bone was analyzed by scanning electron microscopy (Quanta 200 ESEM, FEI Company, Hillsboro, OR, USA) in low vacuum and a semi-hydrated state at 3.0 kV without sputter coating. Morphologies and orientations of the osteocyte lacunae in both *Ahsg*+/+ and *Ahsg*−/− bone were compared.

### Confocal Laser Scanning Microscopy

Samples were stained with a rhodamine-B (Sigma-Aldrich GmbH, Steinheim, Germany) solution diluted in a ratio of 1∶5 with phosphate buffered saline (PBS) for 3 days. After washing with PBS at neutral pH, confocal laser scanning micrographs were obtained with a Zeiss LSM 510 scanning system (Zeiss MicroImaging GmbH, Jena, Germany) equipped with a 100X oil immersion microscope objective having a numerical aperture of 1.4. The excitation wavelength was set to 514 nm, while the emission was measured at a range from 550 up to 650 nm. Image stacks were measured to a penetration depth of 20 µm with each image taken at 0.1 µm intervals. The spatial pixel resolution of each image in the stack was 0.2×0.2 µm. Z-projections of image stacks were obtained with the stddev-method plug-in of the ImageJ image analysis package [36].

### Phase Enhanced X-ray Microradiography

Representative samples from the femur of *Ahsg*+/+ and *Ahsg*−/−, mounted in a custom made tensile tester, were imaged in the BAMline imaging beamline of the Helmholtz Zentrum Berlin für Materialien und Energie. Phase enhanced images were obtained for each sample, tensed until failure, using a partially coherent and monochromatic beam with an energy of 30 KeV. Due to Fresnel propagation and edge enhancement, microstructural details such as defects, cracks, and osteocytes were readily seen. Samples were analyzed for the evolution of strain using customized software (Labview 7.0, National Instruments, Munich, Germany) to analyze strain along the length of the sample.

### Micro-computed Tomography, µCT and Radiography

Three representative samples of sectioned femora of each genotype were measured for their characteristic X-ray attenuation coefficients. Samples were mounted upright on a rotation stage of a µCT laboratory source (Skyscan 1072, Skyscan, Kontich, Belgium) and scanned to obtain radiographs from multiple angular orientations at an energy of 46 keV using 218 µA and acquisition times of 2.8 seconds (pixel resolution was 1.72 µm). Due to the parallelepiped shape of each sample, it was possible to identify and measure radiographs of the front and side projections corresponding to the sample widths and depths. These were used to determine the sample dimensions (for estimates of thickness) as well as the relative absorption of multiple regions along the sample (relative to the regions used as background) using ImageJ [36]. In addition, the sample dimensions were confirmed by scanning electron micrographs of the same samples. Average sample thickness and attenuation were determined from five measurement points randomly collected on the sample within orthogonal sample projections. The sample attenuation was determined with the following equation:
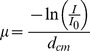
(1)where μ is the attenuation coefficient, I is the intensity of the beam passing through the sample, I_0_ is the empty-beam intensity without the sample (background), and d_cm_ is the thickness of the sample corresponding to the attenuation measured in centimeters (obtained from orthogonal projection).

### Nanoindentation (NI) Measurements

All NI samples were embedded in polymethylmethacrylate (PMMA) and oriented such that the tangential-longitudinal plane was parallel to the indentation surface. The indentation surfaces were polished with 3 and 1 µm grit-sized polishing paper and subsequently, with 0.3 and 0.05 µm sized alumina particle suspensions (AP-D, Struers A/S, Ballerup, Denmark). Polished samples were mounted into a nanoindenter (Ubi 1, Hysitron Inc., Minneapolis, MN, USA) and indentations were performed on the sample’s tangential-longitudinal plane using a Berkovich indenter ∼10 µm^2^. The loading cycle for each indent consisted of 5 s loading, 30 s holding at 5000 µN load, and 5 s unloading. All NI measurements were performed in dry conditions. Each sample was indented a total of 10 indents transverse to the tangential-longitudinal plane.

### Micro-tensile Mechanical Measurements

Micro-tensile experiments were performed with a custom-made micro-tensile testing apparatus with a translation motor (M-126.DG, Physik Instrumente, Karlsruhe, Germany) and a 250 g load cell (ALD-MINI-UTC-250, A.L. Design Inc., Buffalo, NY, USA) at a constant strain rate of 0.2 µm s^−1^ to simulate quasi-static loading. Each sectioned sample was glued to stiff teflon foils with cyanoacrylate (Loctite Deutschland GmbH, Munich, Germany) and mounted into the tensile apparatus such that the tensile axis was parallel to the longitudinal axis of the sample. Strain measurements were accomplished by measuring the percent displacement between optical marks made by a 50 µm tip sized marker (Copic Multiliner, Too Marker Products, Japan) on the sample and tracked by a video camera (Basler A101f, Basler Vision Technologies, Ahrensburg, Germany). Both the camera and the tensile tester were controlled by customized software (Labview 7.0, National Instruments). Each sample was tensed until fracture. Stress-strain behaviors were obtained for each sample measurement and further analysis were performed to determine characteristic materials properties. All samples were kept hydrated during measurements and tensed until failure.

### Polarized Raman Microspectroscopy

A continuous laser beam was focused down to a micrometer size spot on the sample through a confocal Raman microscope (CRM200, WITec GmbH, Ulm, Germany) equipped with piezo-scanner (P-500, Physik Instrumente, Karlsruhe, Germany). The diode pumped linearly polarized 785 nm near infrared laser excitation (Toptica Photonics AG, Graefelfing, Germany) was used in combination with a water immersed 60X (Nikon, NA  = 1.0) microscope objective. For each sample, 10 different points were analyzed. To overcome the orientation dependence of the Raman intensity signal with respect to the orientation of the laser polarization [37], each analyzed point was an average of 4 spectra (total integration time was 10 s) acquired with different polarization orientations (0, −45, 45 and 90 degrees with respect to laboratory coordinates) The spectra were acquired using an air-cooled CCD detector (PI-MAX, Princeton Instruments Inc., Trenton, NJ, USA) behind a grating (300 g mm^−1^) spectrograph (Acton, Princeton Instruments Inc., Trenton, NJ, USA) with a spectral resolution of 6 cm^−1^. ScanCtrlSpectroscopyPlus (version 1.38, WITec GmbH, Ulm, Germany) was used for the experimental setup and spectral processing. Raman intensities of the Amide I band (1600–1700 cm^−1^) and PO_4_^3−^ (910–990 cm^−1^) were obtained by subtracting the respective background intensity and integrating over the wave number regions using a sum filter for each spectrum.

### Scanning Electron Microscopy of Fracture Surfaces

Fracture surfaces of representative samples tensed to fracture were imaged by scanning electron microscopy using a field-emission scanning microscope (LEO FE-SEM Gemini 1550, LEO Electron Microscopy Group, Oberkochen, Germany) at 3.0 kV. Samples were sputter coated with a 3 nm uniform layer of palladium using a sputter coater (Bal-Tec SCD-050 Cool Sputter Coater, Bal-Tec AG, Schalksmuehle, Germany).

### Lab-based Small Angle X-ray Scattering (SAXS)

Samples were measured with a laboratory-based SAXS system (Nanostar, Bruker AXS, Madison, Wisconsin, USA) with copper Kα radiation at 40 kV, 35 mA power settings. Each data point represents three scan frames with an acquisition time of 1 hour. Analysis of the data using Fit2D (AP Hammersley, ESRF, Grenoble, France) produced a T-parameter used to evaluate the thickness of mineral particles in the samples.

### In situ Synchrotron-based SAXS

Micro-tensile measurements were performed by mounting the custom-made microtensile apparatus in beamline ID2 at the European Synchrotron Radiation Facility (ESRF, Grenoble, France). Synchrotron radiation was used to measure the SAXS patterns during microtensile measurements of hydrated samples. Specifically, the meridional stagger D of collagen molecules in the fibril led to an axial diffraction pattern. Percent changes in the positions of the peaks provided measures of fibril strain while percent changes in the positions of marks on the sample surface provided measures of tissue strain. The fibril direction was aligned along the tensile direction in the sample, as seen from the SAXS images of the fibril meridional pattern (Figure S2). Subsequent filtering of off-axis measurements were later determined during the SAXS analysis and not included in the data.

Analysis of data involved radial integration of SAXS images azimuthally. After integration, custom scripts were used for peak fitting routines of the 1^st^ order reflection from the intensity profile of the meridional collagen SAXS pattern. An exponentially modified Gaussian with a background term was used as a fitting function (Figure S2).

The X-ray beam wavelength λ was 0.995 Å and the beam width was ∼200 µm×40 µm high. A FRELON 2000 CCD detector connected to an X-ray image intensifier TTE (TH 49–427, Thomson CSF, Moirans, France) was used for reading the SAXS pattern. A beamstop with integrated diode was used for normalization of the spectra to absolute scattering intensities. SAXS frames were collected concurrently by using SPEC (Certified Scientific Software, Boston, MA, USA) with automatic correction for dark field current. The sample to detector distance for the FRELON 2000 dectector was 10.0 m ±3 mm measured with linear encoder and mechanical measurement [38]. SAXS data frames had 1,024×1,024 pixels and a pixel size of 164.4×164.7 µm^2^.

All samples were tensed to failure at a constant motor velocity of 0.2 µm s^−1^ and SAXS measurements were collected at various points along the stress-strain curve. Exposure time for the frames was 0.1 s. X-ray irradiation of the sample was blocked between exposures.

### Quantitative Backscattered Electron Microscopy

The evaluation protocol of quantitative backscattered signal intensities is based on the work of other groups and has been described previously [39], [40]. The scanning electron microscope (LEO 435 VP, LEO Electron Microscopy Ltd., Cambridge, England) was operated at 15 kV and 665 pA at a constant working distance (BSE Detector, Type 202, K.E. Developments Ltd., Cambridge, England). A pixel size of 3 µm was chosen following the recommendation of Roschger and co-worker [41]. Mineralization profiles were generated of both cancellous bone in the ossification zone and cortical bone of femoral and tibial samples in each case. The degree of mineralization is presented as the mean Ca content (mean Ca-Wt%).

## Results

### Normal Trabecular Bone Mass, but Reduced Length of Long Bones in *Ahsg−/−* Mice

**Figure 1:**
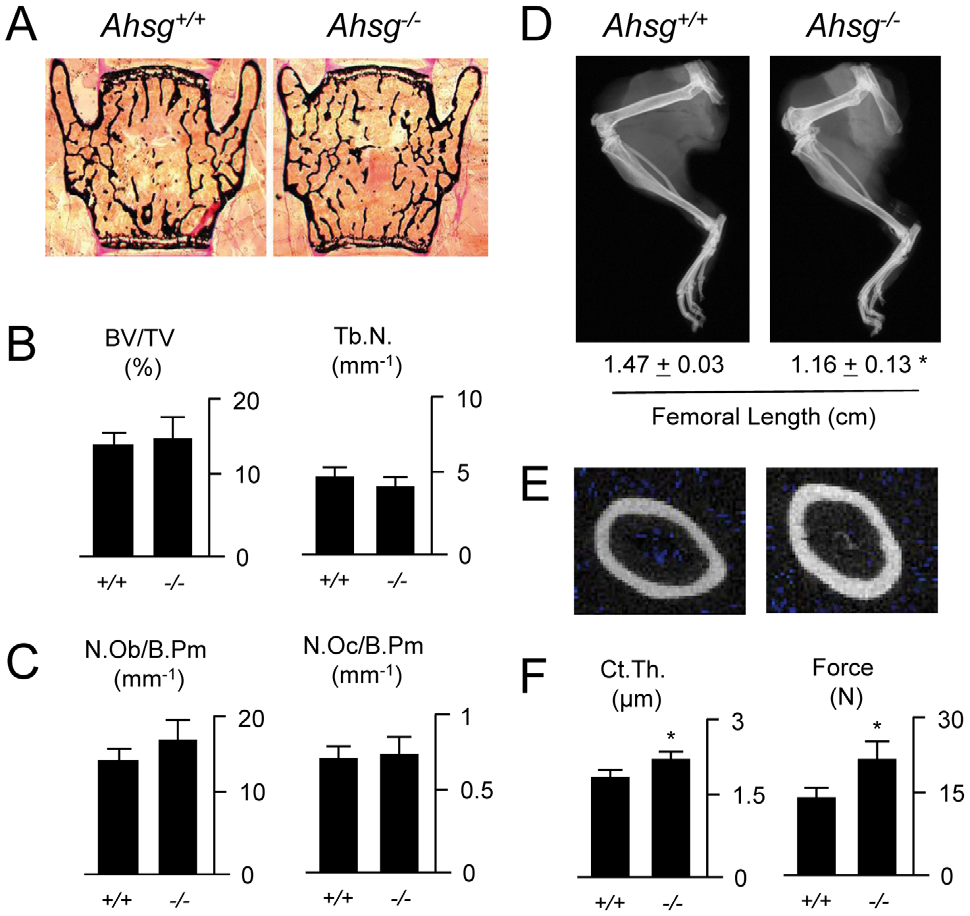
Normal trabecular bone mass, but increased cortical bone strength in fetuin-A deficient mice. (*A*) Von Kossa/van Gieson-stained undecalcified sections of the spine from 4 months old wildtype and fetuin-A deficient mice. (B) Histomorphometric quantification of the trabecular bone volume (BV/TV, bone volume per tissue volume) and the trabecular number (Tb.N.). (C) Histomorphometric quantification of the osteoblast number (N.Ob./B.Pm, number of osteoblasts per bone perimeter) and the osteoclast number (N.Oc./B.Pm, number of osteoclasts per bone perimeter). (D) Contact radiographs of the hindlegs from 4 months old *Ahsg*+/+ and *Ahsg*−/− mice. The femoral length is given below. (E) Cross-sectional µCT scanning of the femora. (F) Quantification of the cortical thickness and the force to failure in three-point-bending assays. All values represent mean±SD (n = 8 per group). Asterisks indicate statistically significant differences (p<0.05).

To analyze whether fetuin-A deficiency would result in impaired bone structure in the absence of soft tissue calcifications, we analyzed the skeletal phenotype of 4 months old *Ahsg*−/− mice that were backcrossed for 10 generations into the genetic background C57BL/6. We first performed non-decalcified histology on the spine followed by static histomorphometry (Fig. 1A). Here we did not observe a significant difference between *Ahsg*−/− mice and wildtype littermates in terms of the trabecular bone volume and the trabecular number (Fig. 1B), thereby demonstrating that the osteopenia observed in *Ahsg*−/− mice on a DBA/2 genetic background [25] was secondary to ectopic calcification. This was further underscored by the fact that we observed no significant difference in the number of bone-forming osteoblasts and of bone-resorbing osteoclasts between wildtype and *Ahsg*−/− littermates (Fig. 1C). We also did not find any gross abnormalities in bone matrix mineralization, since the osteoid volume was not significantly different between the two groups. The length of the spine was also unaffected by the absence of fetuin-A. In contrast fetuin-A deficient long bones, especially the femora were shorter than wildtype bones (Fig. 1D). When we utilized cross-sectional µCT scanning, we further found an increased cortical thickness of fetuin-A deficient femora compared to wildtype controls (Fig. 1E). This led us to perform three-point-bending assays with these bones, where we found that the force to failure was significantly increased for fetuin-A deficient femora (Fig. 1F). Taken together, these results demonstrated that the absence of fetuin-A in long bones improves their biomechanical competence, thus raising the question, whether the intrinsic material properties of cortical bone are altered in *Ahsg*−/− mice.

### Microstructure, Micromechanics as Well as Mineral and Collagen Characteristics of Cortical Bone are Unaffected by the Absence of Fetuin-A

**Figure 2:**
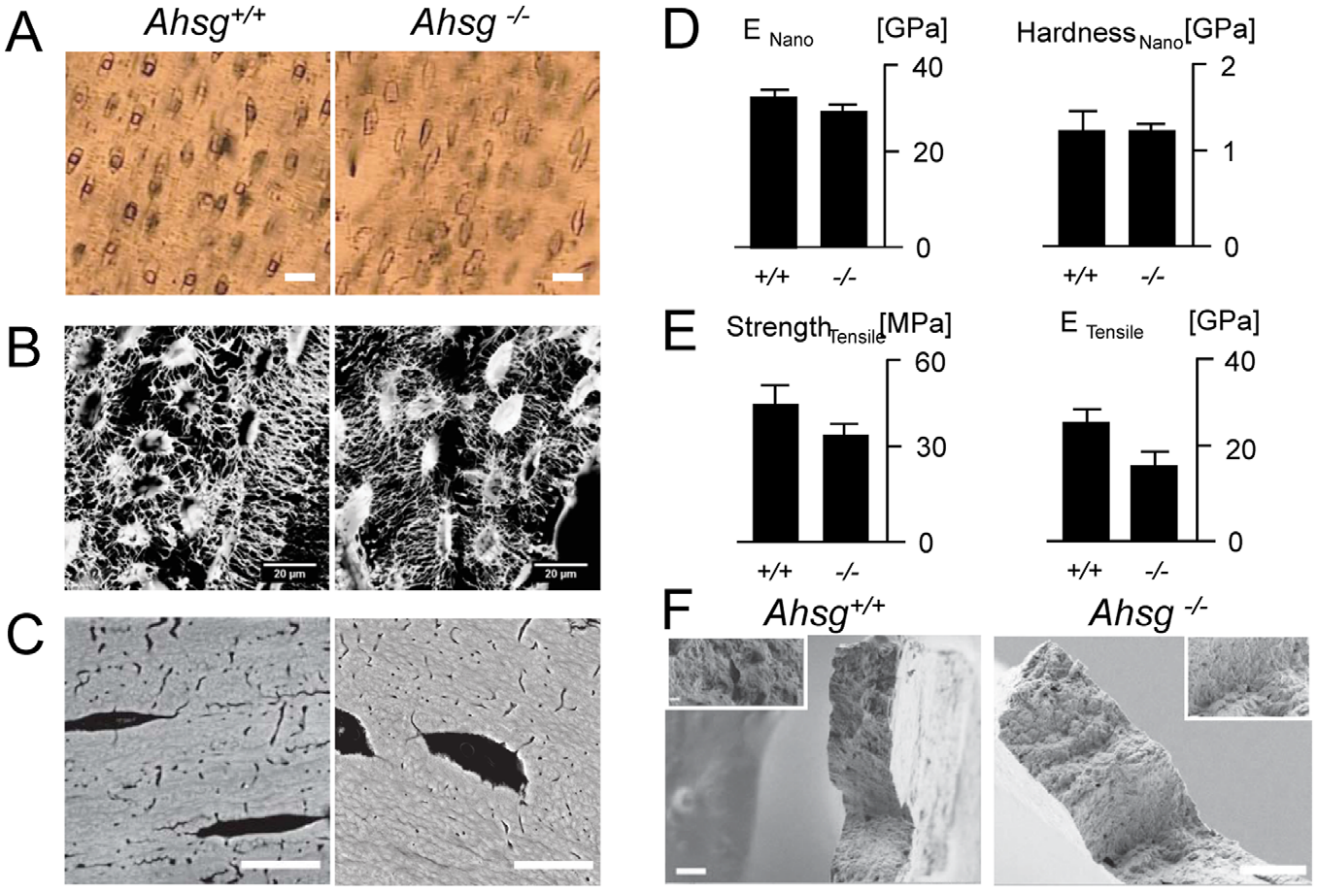
Microstructural and micro-scale mechanical properties of *Ahsg*+/+ and *Ahsg*−/− cortical bone. (A). Light microscopy of osteocyte lacunae in *Ahsg*+/+ and *Ahsg*−/− samples, respectively (scale bar: 20 µm) (B). Laser scanning confocal microscopy using Rhodamine-B as a contrasting agent showing osteocytic and canalicular networks in *Ahsg*+/+ and *Ahsg*−/− samples, light areas are intensely stained with Rhodamine-B, scale bar: 20 µm. (C) Backscatter scanning electron microscopy revealing the microstructure at the surface and no significant differences in density, scale bar: 10 µm. (D) Nanoindentation (n = 80) measurements of the indentation moduli and hardness of *Ahsg*+/+ and *Ahsg*−/− bone samples (E) Micro-tensile (n = 20) measurements of tensile strength and elastic moduli in wildtype and fetuin-A deficient bone samples (F) Representative *Ahsg*+/+ and *Ahsg*−/− fracture surfaces showing evidence of brittle failure, scale bar: 20 µm, inset: higher magnification of the fracture surface, scale bar: 2 µm.

Micro-structural analyses focused on homogenous bone regions from the femoral cortex of adult mice. Typical *Ahsg*+/+ and *Ahsg*−/− samples observed with brightfield light microscopy (Fig. 2A) and confocal laser scanning microscopy (Fig. 2B) revealed no characteristic micro-structural differences. No differences were observed in either the morphology or frequency of osteocyte lacunae nor the canaliculae in both the *Ahsg*+/+ and *Ahsg*−/− samples (Fig. 2A,B). Further complementing observations from optical microscopy, scanning electron microscopy was used to examine the microstructure in the *Ahsg*+/+ and *Ahsg*−/− samples (Fig. 2C). As observed in these various modes of microscopies, both *Ahsg*+/+ and *Ahsg*−/− samples did not display any significant differences in microstructure. Importantly, the micrographs presented in Fig. 2 illustrate that the starting material for micro-mechanical analysis was free of structural defects and preparation artifacts.

Representative samples of the femora from *Ahsg+/+* (n = 4) and *Ahsg−/−* (n = 4) mice were tested by nanoindentation. Figure 2D shows that the elastic moduli of wildtype samples were found to be 32.5±0.8 GPa (mean ± SEM), whereas the fetuin-A deficient samples were found to be 29.0±0.8 GPa. The respective hardness values were 1.26±0.06 GPa in wildtype and 1.26±0.04 GPa in fetuin-A deficient bone (Fig. 2D). Thus, both the indentation moduli and hardness values were not significantly different between wildtype and fetuin-A deficient bone.

In addition, samples from *Ahsg+/+* and *Ahsg−/−* mice (n = 4 in each case) were measured under uni-axial tension until failure in physiologically wet conditions. A total of 10 replicate measurements for each genotype were measured. Ultimate tensile strength (UTS) values of *Ahsg+/+* and *Ahsg−/−* samples were found to be 44.5±6.2 MPa and 34.4±3.8 MPa, respectively. Figure 2E illustrates that the elastic moduli of *Ahsg+/+* samples were 25.4±3.2 GPa (mean ± SEM) and 16.2±3.1 GPa for *Ahsg−/−* samples. Like the nanoindentation measurements reported in Fig. 2D, the tensile moduli and UTS of wildtype *and* and fetuin-A deficient bone samples were not statistically different from each other (*p* = 0.11 and *p* = 0.33 for tensile modulus and UTS, respectively) suggesting that the bone materials properties of these bone samples were very similar at the micro-scale. Figure 2F shows representative fractured samples from micro-mechanical tensile measurements examined by scanning electron microscopy to visualize the fracture surfaces. Samples from both *Ahsg+/+* and *Ahsg−/−* mice showed virtually indistinguishable fracture surfaces, which are typical of brittle fractures. At higher magnifications, both *Ahsg+/+* and *Ahsg−/−* bone showed collagen fiber bundles with similar orientations and structure (Fig. 2F inset). Indeed, micro-mechanical testing validates that the bone material in wildtype and *Ahsg*−/− mice is very similar despite the clear difference at the macro-scale in mechanical strength and the slightly elevated mineral content of whole fetuin-A deficient bone (Fig. S3).

**Figure 3:**
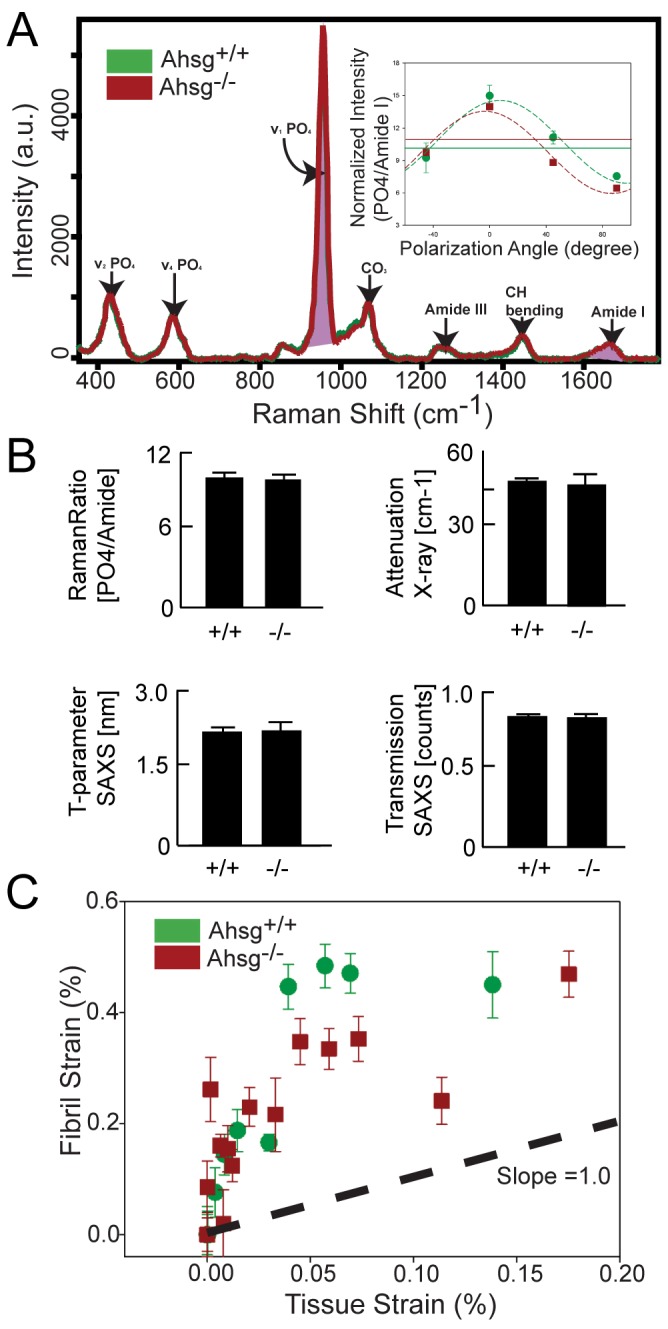
Mineral and organic components in *Ahsg*+/+ and *Ahsg*−/− femoral cortical bone. (A) A typical Raman spectrum of representative *Ahsg*+/+ and *Ahsg*−/− samples with peaks at 910–990 cm^−1^ and 1600–1700 cm^−1^ representing PO_4_^3−^ (mineral) and Amide I (organic matrix) groups, respectively. inset: Normalized intensity measurements at polarization angles of −45, 0, 45, 90 were made to address the orientation artifacts of Raman intensity of type I collagen for both *Ahsg*+/+ and *Ahsg*−/−. The dashed lines are fits which estimate parameters characteristic of sample orientation and mineralization. The solid lines indicate mean intensity values of mineralization in the samples. (B) The mineral content normalized with the organic matrix can be observed by Raman ratios between *Ahsg*+/+ and *Ahsg*−/− samples. Further complementing these observations, measurements of the mineral component were made with X-ray attenuation, absorption, as well as small-angle X-ray scattering. (C) Comparing fibrillar versus tissue strains in *Ahsg*+/+ (green circles) and *Ahsg*−/− (red squares) bone. Samples were measured with in-situ synchrotron small angle X-ray scattering to determine the amount of strain contributed by the collagenous fibrils within the *Ahsg*+/+ and *Ahsg*−/− samples. Dashed lines represent orientation guides.

Polarized Raman micro-spectroscopy was performed to determine the chemical composition of cortical bone to reveal differences in organic to mineral compositions as shown in Fig. 3A [37]. Absolute intensities of the mineral peak (PO4^3−^) were normalized to intensities of the Amide I peak to obtain a normalized ratio of mineral to organic in each sample. The mean relative ratio was 9.80±0.194 for *Ahsg+/+* samples and 9.56±0.27 for the *Ahsg−/−* samples, respectively (Fig. 3B). ANOVA statistical evaluation of the ratios between the *Ahsg+/+* and *Ahsg−/−* sample groups showed no statistically significant differences (P = 0.46 for the PO_4_/Amide I).

To further validate the degree of mineralization found in Raman spectroscopy, X-ray attenuation and absorption experiments were performed to compare the differences between fetuin-A deficient and wildtype samples (Fig. 3B). Micro-computed tomography revealed for *Ahsg+/+* samples an attenuation coefficient of 47.0±1.7 cm^−1^ (mean ± SEM), whereas *Ahsg−/−* samples had a mean value of 46.0±3.2 cm^−1^. Judged by one-way ANOVA statistics, the attenuation coefficients of *Ahsg+/+* and *Ahsg−/−* samples were not significantly different (*p* = 0.36). Likewise in a laboratory based X-ray scattering experiment, the X-ray beam absorption, which is a measure of X-ray density was similar in *Ahsg+/+* and *Ahsg−/−* samples. Additional analysis provided a T-parameter derived from the scattering data to describe the dimensions of the mineral crystallites in the *Ahsg+/+* and *Ahsg−/−* samples. The T-parameter derived from the scattering data were very similar in that 2.15 nm ±0.05 were calculated for *Ahsg+/+* samples and 2.19 nm ±0.09 for the *Ahsg−/−* samples (n = 10, *p* = 0.06).

Using in situ micro-tensile measurements coupled with synchrotron diffraction, the behavior of the mineralized collagen fibrils was examined with respect to the deformation occurring at the tissue level. In the SAXS regime of reciprocal space, the D period of collagen molecules in the fibril was measured and the deformation in each sample was tracked by following the positions of the diffraction peaks which allowed for a measurement of the collagen fibril strain. When normalized with respect to the tissue strains of the sample, we found little differences in strain between the populations of *Ahsg+/+* and *Ahsg−/−* samples (Fig. 3C). We observed that the tissue strains in both *Ahsg+/+* and *Ahsg−/−* samples occurred within a small range of 0–0.2% before failure. No plastic deformation was observed in either sample types before failure. The normalized fibril-tissue strains of mouse bone samples regardless of the *Ahsg* genotype were usually 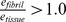. This suggested that deformation behaviors of the fetuin-A deficient and wildtype mice samples were similar.

We did not detect differences in the microstructure of fetuin-A deficient bone that would explain the increased mechanical strength determined by macro-scale mechanical testing. The striking length difference between wildtype and *Ahsg−/−* bones could also not be explained by microscale compositional differences. Failure to detect clear differences in bone cellularity effectively ruled out bone cell dysregulation as a likely cause for the shorter femora. Likewise the differences in total bone mineral could not be accounted for by the microscale composition determined by the aforementioned X-ray and Raman spectroscopic measurements.

### Increased Mineral Content in the Growth Plates of Fetuin-A Deficient Long Bones

**Figure 4:**
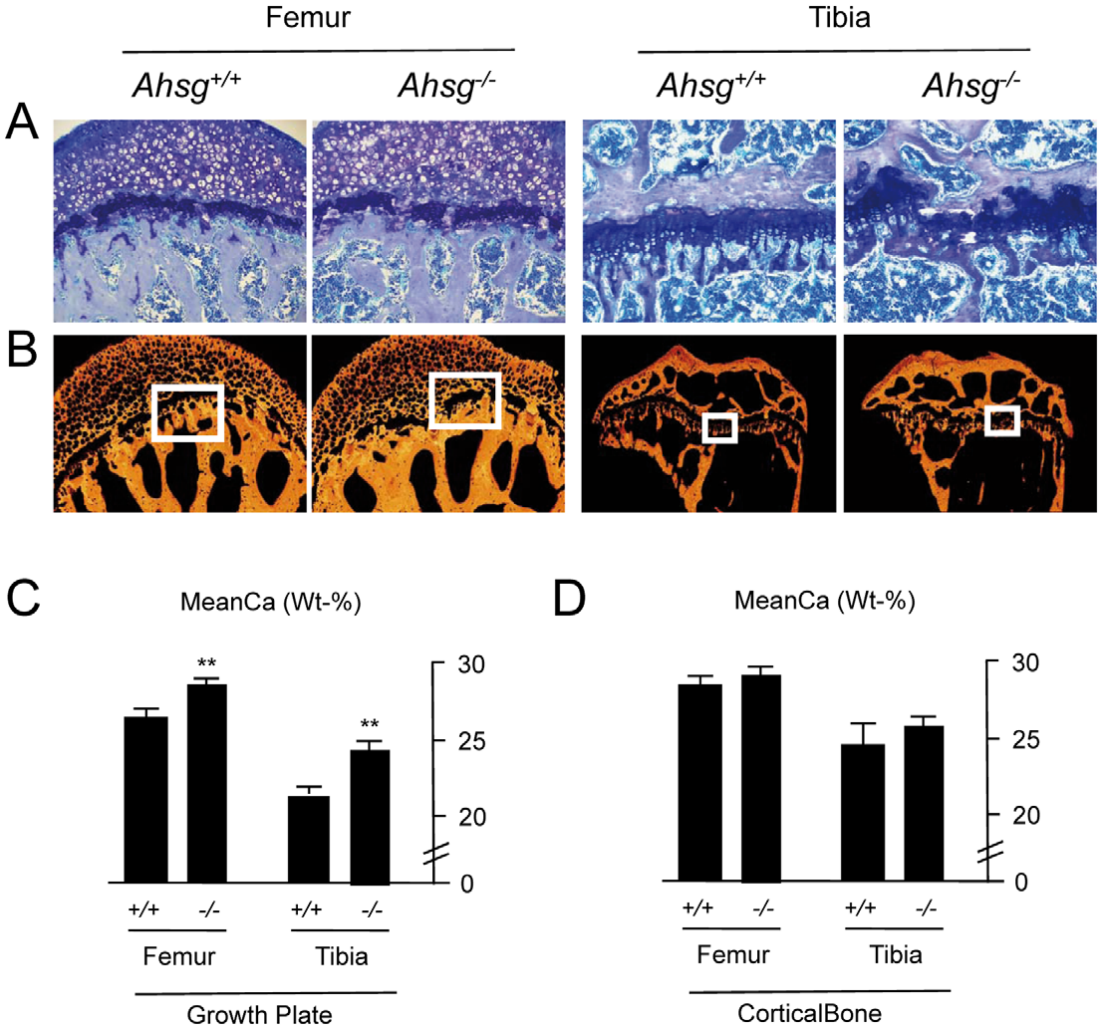
Growth plate morphology and mineralization in *Ahsg*+/+ and *Ahsg*−/− mice. (A) Wildtype mice had dark blue stained cartilage cores within metaphyseal trabeculae of the growth plates, whereas *Ahsg*−/− had completed the cartilage-to-bone transition without the remains of cartilage cores within the trabeculae. Moreover, *Ahsg* −/− mice showed pronounced discontinuities in the chondrocyte column organization in comparison to the wildtype mice (Toluidine blue staining). (B) *Ahsg* −/− mice frequently showed thickened calcified bridge formations across their growth plates, which was confirmed by backscattered electron microscopy. The orange and black areas correspond to mineralized and non-mineralized tissue, respectively. (C) The mineral content in *Ahsg*−/− mice was significantly increased in both tibial and femoral growth plates in comparison to wildtype mice as judged by quantitative backscattered electron imaging. (D) In contrast, the mineralization (mean Ca Wt%) of the femoral and tibial cortices was similar in *Ahsg*−/− and *Ahsg*+/+ mice.

Since a reduced femoral length can also explain the increased biomechanical competence in bending of fetuin-A deficient femora, we next analyzed the growth plate cartilage in these mice. The ossification zone in growth plates of wildtype tibia and femora shown in Fig. 4A revealed the appearance of cartilage cores in the primary and secondary trabeculae indicating a non-completed cartilage-to-bone transition at age 4 months. This is in contrast to the *Ahsg*−/− growth plates, which showed a completed bone mineralization without the occurrence of cartilage cores within the trabeculae. Similarly the analysis of *Ahsg*−/− distal femur growth plates revealed more mineralized bridges per growth plate than wildtype (4.0±1.4 vs. 1.6±0.5, n = 6, p = 0.009) suggesting more complete growth plate mineralization and closure (Fig. S4). Furthermore, differences between wildtype and *Ahsg−/−* mice were obvious with regard to the growth plate chondrocyte organization (Fig. 4A). *Ahsg−/−* mice had discontinuities across the growth plates and regions of thickened calcified bridge formations (Fig. 4A) as observed by backscattered electron microscopy from the bisection in mineralized and non-mineralized tissue (Fig. 4B). The quantification of backscattered signal intensities showed that the mineral content in *Ahsg−/−* mice was significantly increased in both tibial and femoral growth plates in comparison to *Ahsg+/+* mice (Fig. 4C). This difference between *Ahsg−/−* and *Ahsg+/+* mice was restricted to the measured calcified growth plates since the cortical counterparts showed no comparable difference in the degree of mineralization (Fig. 4D). Focusing on the inter-site differences in femoral and tibial regions of interest both *Ahsg−/−* and *Ahsg+/+* showed increased calcium content in the femoral growth plates and cortices (Fig. 4 C,D).

In summary, the observed increase in macroscale mechanical bending strength of the fetuin-A deficient bone was most likely due to stunted growth caused by increased or premature growth-plate cartilage mineralization. This also corresponds to the associated overall shorter length, increased cortical thickness and slightly increased bone diameter at the midshaft position found in the skeletal tissues. Collectively, these changes resulted in macroscopically stronger femora and tibia in fetuin-A deficient mice than in wildtype adult mice, despite similar microscopic properties of the bone material.

## Discussion

Fetuin-A/*Ahsg* content had little effect on bone mineral or matrix quality in the cortex, instead it dramatically affected the growth plate morphology. Femora from fetuin-A deficient mice were approximately 30% shorter than in age-matched wildtype mice (Fig. 1). This phenotype of *Ahsg−/−* mice signifies a form of mineralization dependent dysplasia that had previously gone unnoticed. An earlier study of *Ahsg−/−* mice maintained on a mixed genetic background of 129SvJ/B6, likewise reported shorter long bones in the null mutants [24], but missed two features presented here. Firstly, we determined that the foreshortening was associated with increased mineralization restricted to the growth plate chondrocytes. Additionally, the remainder of the bone material was structurally and mechanically equivalent to wildtype bone. Here, we reconcile the fetuin-A deficient bone phenotype with the well-established role of fetuin-A as a potent systemic inhibitor of mineralization [9], [11], [13], [15], [20], [25], [27], [42], [43] and offer a simple, straightforward explanation for the observed long bone growth defect–we propose that the stunted growth is a consequence of premature growth plate mineralization. The role of fetuin-A activity as a regulator of mineralization of hypertrophic chondrocytes in the growth plate fits well with its tissue distribution. Growth plate cartilage is free of fetuin-A, because it is not vascularized. Vascularization and blood supply enter at a time when mineralization commences, a process highly controlled by molecular inhibitors to regulate the kinetics of mineralization. Any change in fetuin-A concentration at this critical time translates into noticeable changes in mineralization in solution, a biochemical process studied in great detail by us [8], [9], [11], [44]–[47] and others [10], [12], [14], [15], [29], [30], [48], [49]. Endochondral ossification represents the culmination of a sequence of changes in the cartilage cells and their associated matrix that must always occur in the same order and requires a minimum period of time [50]. The developmental regulation of the growth plate progresses from the condensation of mesenchymal cells, their differentiation into chondrocytes and finally maturation to matrix-secreting hypertrohic chondrocytes that cross-talk to the bone collar and attract blood vessels. Ultimately the hypertrophic chondrocytes undergo apoptosis and mineralize [51]. Our results indicate that the lack of fetuin-A, a serum borne inhibitor of mineralization may accelerate the final step of this process, namely matrix mineralization, thus prematurely terminating growth plate activity and longitudinal bone growth. It is presently unclear, why only the proximal long bones are shorter. There is however, a striking phenocopy of fetuin-A deficieny. Physeal obliteration in humans [52] and the so-called hyena disease in calves [53] are caused by vitamin A-induced premature closure of epiphyses that cause preferential foreshortening of hind limb bones. Vitamin A/retinoic acid is thought to accelerate the progression of chondrocytes from the proliferative phase to the calcifying stage bypassing hypertrophy and thus bone length growth. A role of fetuin-A in the regulation of vitamin A bioavailability and activity merits further study.

Fetuin-A and the human homologue, α_2_-HS glycoprotein, induce alkaline phosphatase in epiphyseal growth plate chondrocytes [54]. The induction of this maturation marker may represent an adaptive response in the presence of surplus mineralization inhibitor, triggering the natural chondrocyte progression into mineralized cartilage. In fact, the growth plate chondrocytes may be the cell type that is physiologically most prone to pathological mineralization, because it grows in an environment well protected from mineral supply and thus mineralization, yet becomes quickly exposed to a mineralizing fluid once the cartilage is vascularized. It will be interesting to revisit the influence of fetuin-A on physiological chondrocyte mineralization in a similar way previously performed with primary osteoblasts [8] and smooth muscle cells [42] as models of physiological and pathological mineralization, respectively.

Our results show that fetuin-A has a developmental rather than a structural role in murine bone. Fetuin-A is known to hinder the precipitation of calcium phosphate in blood serum and subsequently, prevents ectopic calcification of soft tissues. Clearance of fetuin-A containing protein-mineral complexes by the monocytic phagocyte system efficiently prevents local depostion [55]. Patients suffering from chronic kidney disease show decreased free serum fetuin-A and increased serum fetuin-A mineral complexes [17], which can be quantified by a nanoparticle-based serum test measuring overall calcification inhibition [56]. The influence of fetuin-A in initial bone mineralization seems, however, minimal in the present model, despite the fact that fetuin-A is deposited in mineralized bone in large amounts [4]–[7].

## Supporting Information

Figure S1 **Schematic of sample preparation.** (A). Femora of mice were sectioned along the anteriorposterior axis (as seen by micro-CT) scale bar: 3 mm (B). Cortical bone from the mid-diaphysis of each sample were further sectioned (C). Sectioning and polishing along the longitudinal-tangential plane (D). Laser microdissection was used to further section the sample into dimensions of 2.5 mm×0.05 mm×0.15 mm (as seen by phase enhanced X-ray radiography) scale bar: 75 µm.
(TIF)

Figure S2 **Determining the fibrillar strain from SAXS measurements.** (A) Image of the meridional collagen SAXS pattern from Fit2D (B) Radial integration of the meridional collagen pattern produces an intensity profile with respect to Q-space showing the 1st, 2nd, and 3rd order reflections (C) Fibrillar strain can be measured from the percent change in position of the 1st order peak as reflected in the SAXS patterns during in situ tensile measurements.
(TIF)

Figure S3 **Tibia morphology and mineralization in wildtype (** ***Ahsg*** **+/+) and fetuin-A deficient (** ***Ahsg*** **−/−) mice.** The mean calcium content in tibias of *Ahsg* −/− mice was increased in comparison to wildtype mice as judged by quantitative backscattered electron imaging of the enlarged areas shown in the middle panels. Ash weight of tibia halves (shown in the top panels) was also increased in *Ahsg* −/− mice compared to wildtype mice. n = 4; * p<0.05; ** p<0.01.
(TIF)

Figure S4 **Distal femur growth plate morphology and mineralization in wildtype (A, C, E) and fetuin-A deficient (B, D, F) mice.** These representative samples show higher numbers of bone bridges and more mineralization in the growth plate of fetuin-A deficient mice than wildtype mice.
(TIF)
