# Supplementary figures and images for: Accelerated Growth Plate Mineralization and Foreshortened Proximal Limb Bones in Fetuin-A Knockout Mice
Source: PLoS One. 2012 Oct 16;7(10):e47338. doi: 10.1371/journal.pone.0047338 (PMC3473050; doi:10.1371/journal.pone.0047338)

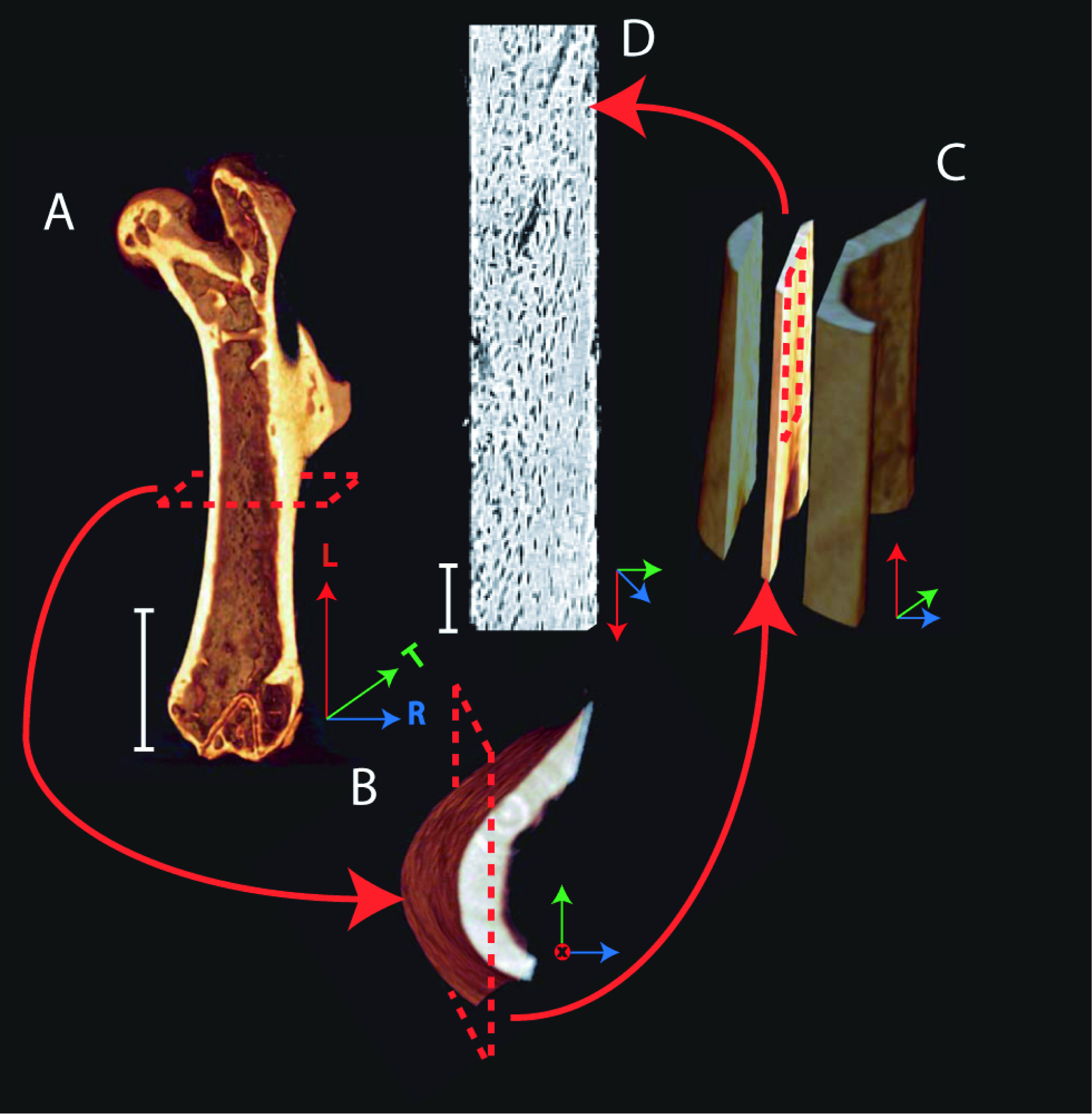

Supplement: Figure S1 — Schematic of sample preparation. (A). Femora of mice were sectioned along the anteriorposterior axis (as seen by micro-CT) scale bar: 3 mm (B). Cortical bone from the mid-diaphysis of each sample were further sectioned (C). Sectioning and polishing along the longitudinal-tangential plane (D). Laser microdissection was used to further section the sample into dimensions of 2.5 mm×0.05 mm×0.15 mm (as seen by phase enhanced X-ray radiography) scale bar: 75 µm. (TIF) [file pone.0047338.s001.tif]

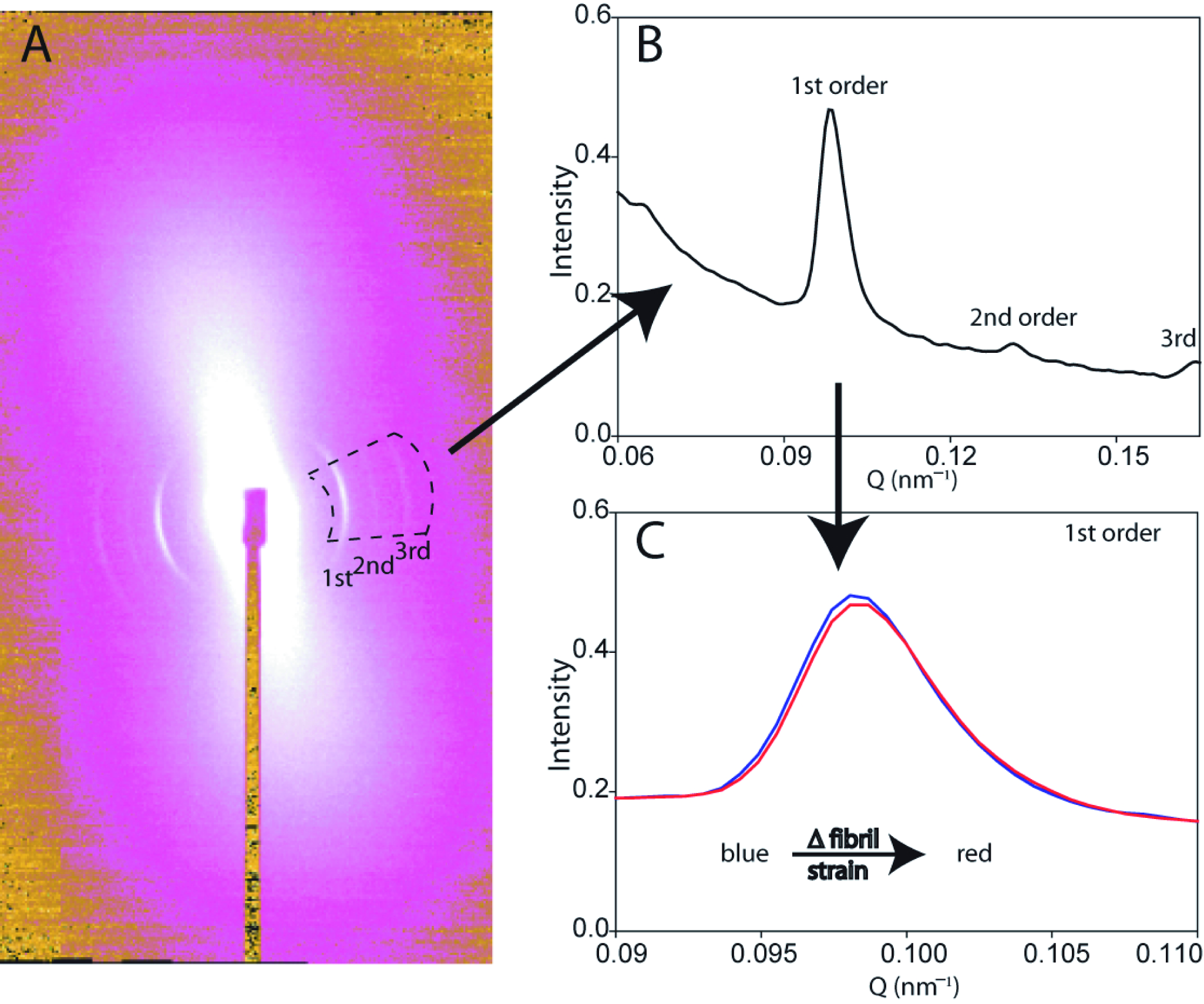

Supplement: Figure S2 — Determining the fibrillar strain from SAXS measurements. (A) Image of the meridional collagen SAXS pattern from Fit2D (B) Radial integration of the meridional collagen pattern produces an intensity profile with respect to Q-space showing the 1st, 2nd, and 3rd order reflections (C) Fibrillar strain can be measured from the percent change in position of the 1st order peak as reflected in the SAXS patterns during in situ tensile measurements. (TIF) [file pone.0047338.s002.tif]

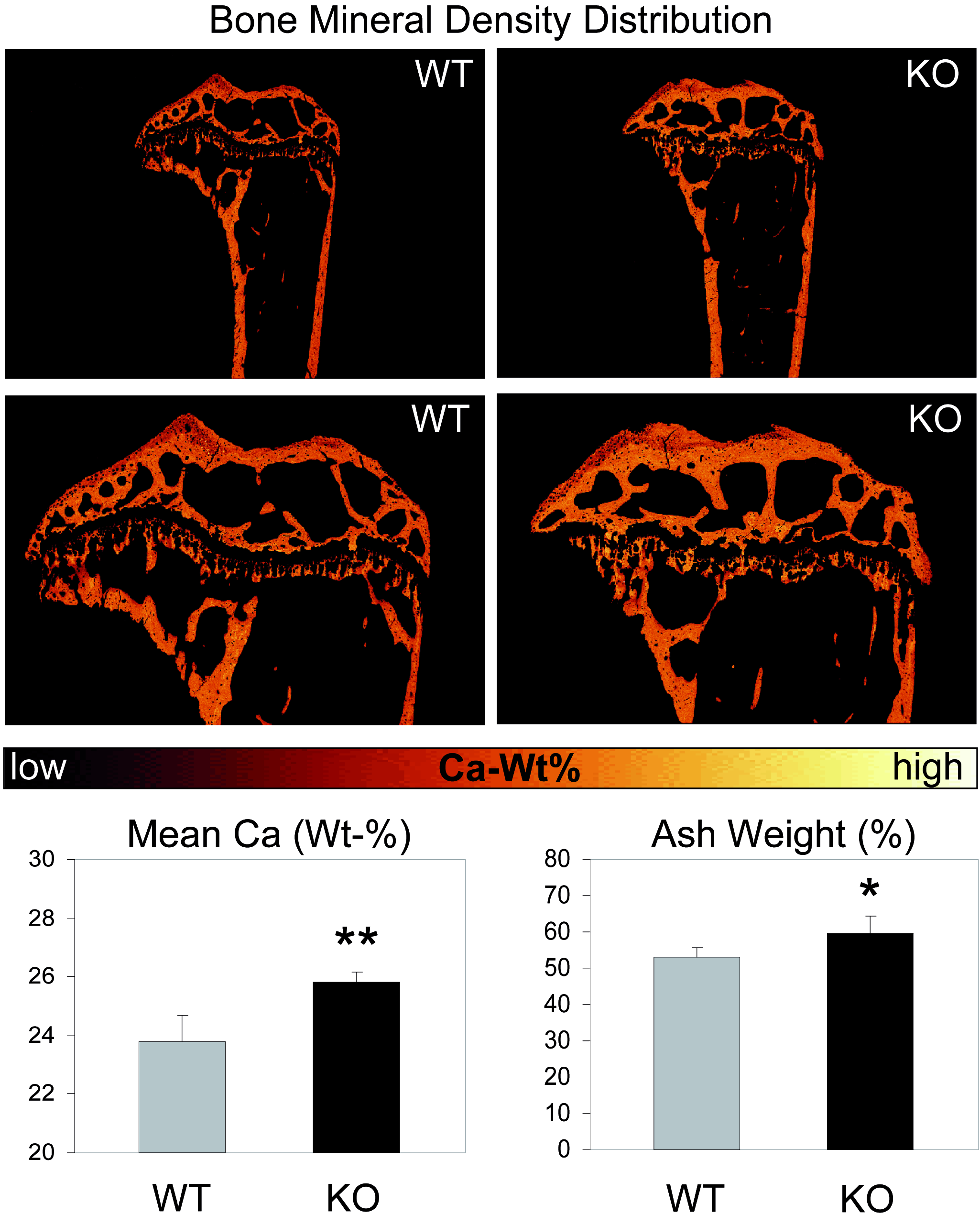

Supplement: Figure S3 — Tibia morphology and mineralization in wildtype ( Ahsg +/+) and fetuin-A deficient ( Ahsg −/−) mice. The mean calcium content in tibias of Ahsg −/− mice was increased in comparison to wildtype mice as judged by quantitative backscattered electron imaging of the enlarged areas shown in the middle panels. Ash weight of tibia halves (shown in the top panels) was also increased in Ahsg −/− mice compared to wildtype mice. n = 4; * p<0.05; ** p<0.01. (TIF) [file pone.0047338.s003.tif]

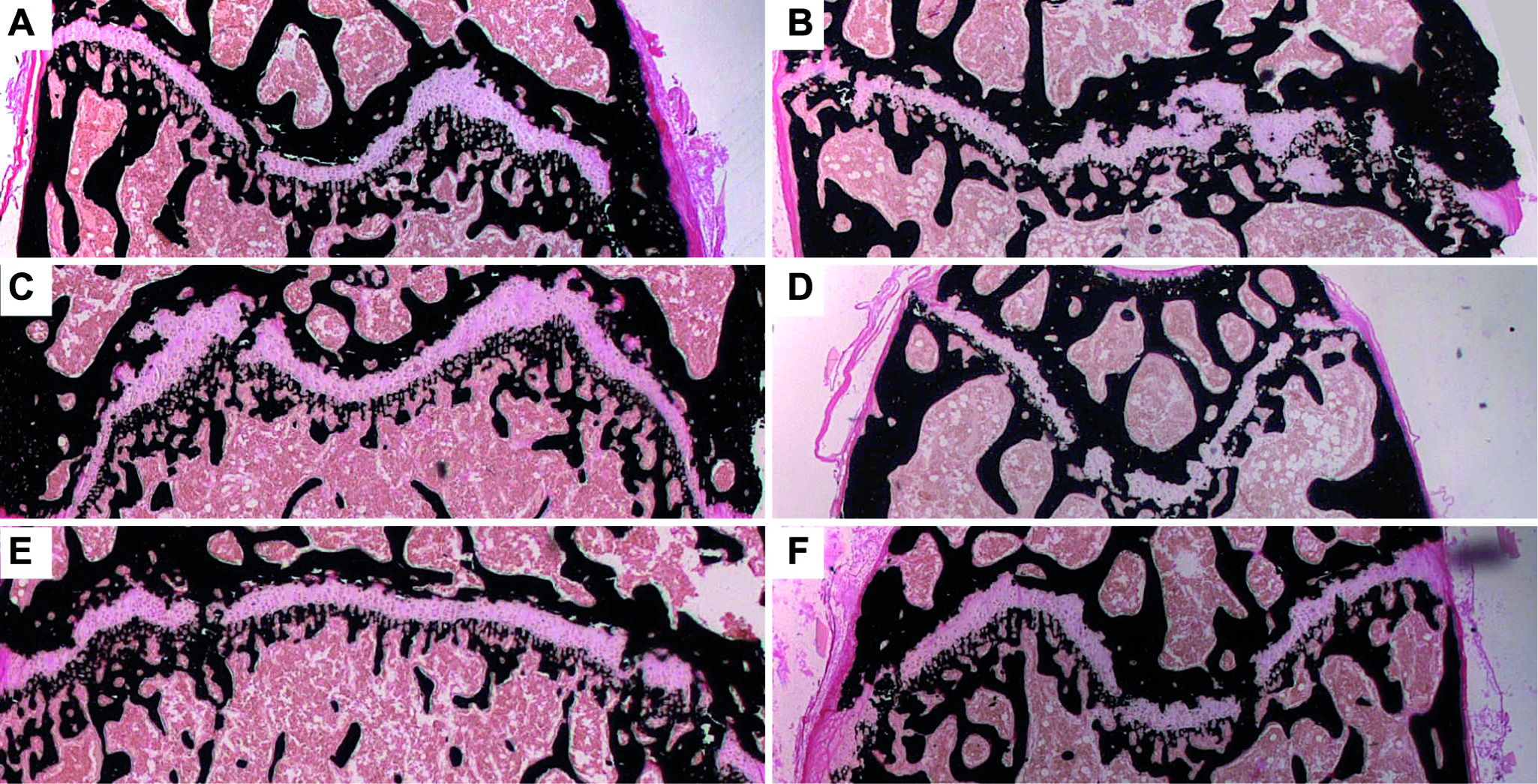

Supplement: Figure S4 — Distal femur growth plate morphology and mineralization in wildtype (A, C, E) and fetuin-A deficient (B, D, F) mice. These representative samples show higher numbers of bone bridges and more mineralization in the growth plate of fetuin-A deficient mice than wildtype mice. (TIF) [file pone.0047338.s004.tif]
